# Relationship of Knee Abduction Moment to Trunk and Lower Extremity Segment Acceleration during Sport-Specific Movements

**DOI:** 10.3390/s24051454

**Published:** 2024-02-23

**Authors:** Mitchell Ekdahl, Sophia Ulman, Lauren Butler

**Affiliations:** 1 Scottish Rite for Children, Frisco, TX 75034, USA; sophia.ulman@tsrh.org; 2 Department of Orthopaed Surgery, University of Texas Southwestern Medical Center, Dallas, TX 75390, USA; 3 Nicole Wertheim College of Nursing and Health Sciences, Florida International University, Miami, FL 33199, USA; lbutler@fiu.edu; 4 Nicklaus Children’s Hospital, Miami, FL 33155, USA

**Keywords:** wearable sensors, motion analysis, sports medicine, biomechanics, knee injury

## Abstract

The knee abduction moment (KAM) has been identified as a significant predictor of anterior cruciate ligament (ACL) injury risk; however, the cost and time demands associated with collecting three-dimensional (3D) kinetic data have prompted the need for alternative solutions. Wearable inertial measurement units (IMUs) have been explored as a potential solution for quantitative on-field assessment of injury risk. Most previous work has focused on angular velocity data, which are highly susceptible to bias and noise relative to acceleration data. The purpose of this pilot study was to assess the relationship between KAM and body segment acceleration during sport-specific movements. Three functional tasks were selected to analyze peak KAM using optical motion capture and force plates as well as peak triaxial segment accelerations using IMUs. Moderate correlations with peak KAM were observed for peak shank acceleration during single-leg hop; peak trunk, thigh, and shank accelerations during a deceleration task; and peak trunk, pelvis, and shank accelerations during a 45° cut. These findings provide preliminary support for the use of wearable IMUs to identify peak KAM during athletic tasks.

## 1. Introduction

Anterior cruciate ligament (ACL) injuries are one of the most commonly reported knee injuries in young athletes, with the highest rates in those who participate in high-risk sports that involve jumping, cutting, and pivoting [1,2]. Given the high prevalence of ACL injuries in this population, screening tools that identify athletes’ risk for injury have been established [3,4,5,6]. These tools are typically designed to identify lateral trunk deviation, medial knee collapse, decreased knee flexion range of motion, and altered hip strategies, as these movement patterns have been reported as risk factors during landing and change-of-direction tasks [7,8,9,10,11,12,13]. Three-dimensional (3D) motion analysis laboratory studies have identified high knee abduction moment (KAM) as a significant predictor of ACL injury risk during landing and change-of-direction movements [11,14,15,16]. A prospective study by Hewett et al. identified increased KAM during a drop vertical jump to be a predictor of ACL injury, with a sensitivity of 78% and specificity of 73% [9]. A more recent in vitro study corroborated these findings with increased KAM leading to increased ACL strain [17]. Similarly, several studies have found an association between high knee abduction moments during cutting maneuvers and increased ACL injury risk [14,15,16]. However, given the cost and significant time demands associated with collecting 3D kinematic and kinetic data, lower-cost and more time-efficient alternatives have been explored [9,18,19,20]. Two-dimensional (2D) video-based tools have been found to be reliable and valid alternatives to assess risk factors of ACL injury during landing and change-of-direction maneuvers, although these tools present challenges for on-field assessments [5,6,21].

Wearable wireless inertial measurement units (IMUs) contain a three-dimensional accelerometer, gyroscope, and magnetometer, and can be mounted on body segments to record segment acceleration, angular velocity, and global orientation. Recently, they have been explored as low-cost, quantitative solutions that can be used on the field for the assessment of injury risk [22]. Pratt et al. reported a moderate positive correlation between peak knee extensor moment and peak thigh angular velocity, derived from IMUs placed at the shank and thigh, during a single-leg landing task in participants after ACL reconstruction [22,23,24,25]. Furthermore, the same author team reported that angular velocity ratios derived from a single IMU sensor were capable of detecting asymmetrical knee loading during a single-leg loading task in participants after ACL reconstruction, highlighting the clinical utility of these sensors [25]. Additionally, Jones et al. placed IMUs at the shank and thigh during landing and change-of-direction tasks and reported a strong positive correlation between knee range of motion and the area under the tibia angular velocity curve, which indicates angular displacement of the tibia [24]. Jones et al. also reported strong correlations between knee range of motion, change in knee flexion moment and knee stiffness, and the area under the tibia acceleration curve during a single leg drop jump landing. Finally, Dowling et al. reported significant correlations between coronal angular velocities derived from thigh and shank IMUs and KAM during a double-leg and single-leg drop jump [23].

Although angular velocity has been shown to be a useful measure for assessing injury risk, gyroscopes are highly susceptible to constant bias, noise, and bias instability during high-impact and high range-of-motion movements [26]. As a result, angular velocity measurements often require the implementation of compensation or calibration techniques to improve measurement accuracy [27,28]. By contrast, accelerometer signals are only affected by noise during highly dynamic movements and therefore require a simple lowpass filter for accurate measurement. Acceleration can easily be extracted from one IMU placed on one body segment, making it a cost- and time-efficient measure. However, there is currently a gap in the literature related to the use of acceleration data for injury risk assessment. Specifically, the relationship between linear acceleration at the trunk and lower extremities and KAM during athletic tasks have not been explored. Thus, the purpose of this pilot study was to assess the relationship between acceleration at the trunk, pelvis, thigh, and shank derived from wireless accelerometers and KAM during sport-specific movements.

## 2. Materials and Methods

### 2.1. Participants

A convenience sample of thirteen healthy participants (26.0 ± 3.4 years, 7 female) were included in this pilot study and completed a single visit in a motion capture laboratory. To be eligible, participants were required to have no orthopedic condition or prior injury (within the past six months) that would limit their ability to perform the required tasks. This study was approved by the host institution’s Institutional Review Board, and all participants provided informed written consent prior to initiating testing procedures. For testing, participants were asked to wear comfortable attire and their personal athletic footwear.

### 2.2. Setup and Equipment

**Figure 1:**
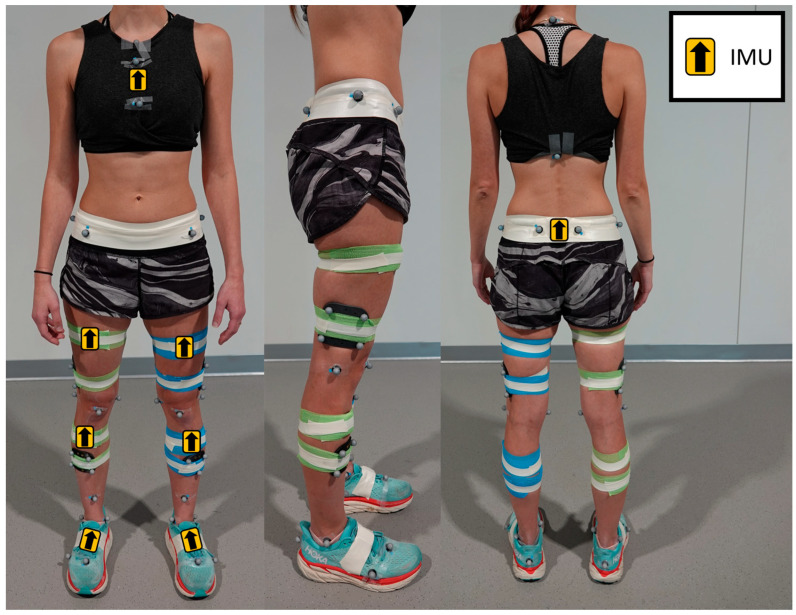
Placement of eight Delsys Trigno Avanti IMU sensors: sternum, sacrum, anterior thighs, anterior shanks, and the dorsal side of the feet. Retroreflective markers are placed on bony landmarks based on a modified Cleveland Clinic marker set [30].

Acceleration data were collected using eight wireless Delsys Trigno Avanti IMU sensors (Delsys Inc., Natick, MA, USA) placed directly on the skin on the body segments of interest, as displayed in Figure 1, and secured with elastic wrap and athletic tape to minimize skin artifact noise in the IMU signal. Specifically, placements included the sternum (midpoint between xiphoid process and jugular notch), sacrum (midpoint between posterior superior iliac spines), anterior thighs (rectus femoris muscle bellies), and the anterior shanks (tibialis anterior muscle bellies) as shown in prior work [28]. Two sensors were also placed on the dorsal side of the feet (along the shoelaces); however, these were primarily used to measure ankle joint kinematics for a separate study [28]. Additionally, the acceleration data were determined to contain excessive noise, likely due to motion artifacts during foot strikes, and were therefore not included in the analyses of this current study. Tri-axial accelerometers (±16 g) in each IMU sensor were used to collect acceleration data at 240 Hz. Data from the gyroscope and magnetometer in each sensor were not used for this study.

Kinematic data was collected with a 14-camera motion capture system (Vicon Motion Systems Ltd., Denver, CO, USA) sampling at 240 Hz [29]. A modified Cleveland Clinic marker set [30] was used to place retroreflective markers on bony landmarks of the trunk, pelvis, and lower extremities, including clusters on the lateral thighs and anterior shanks (Figure 1). Kinetic data were collected with six AMTI force plates embedded in the floor (Advanced Mechanical Technology, Inc., Watertown, MA, USA), sampling at 2880 Hz [29]. All motion capture and accelerometer data were collected with Vicon Nexus 2.11 software and time-synchronized with a Delsys Trigger Module (Delsys Inc., Natick, MA, USA).

### 2.3. Testing Procedure

To begin data collection, a static calibration trial was collected, followed by gait. Kinematic variables from the gait trial were used to confirm accurate marker placement and adjust placement as needed. Participants completed three functional tasks for each leg: single-leg hop, deceleration, and a 45° run cut. Verbal instructions for each task are listed in Table 1. For the single-leg hop task, the subject began on one leg facing the force plates. They then hopped forward as far as possible and landed within the bounds of the force plates on the same leg while maintaining balance for at least 3 s after landing. If the subject did not land within the bounds of the force plates, the starting position was adjusted as necessary, and the task was repeated. For the deceleration task, the subject began approximately 12 feet away from the force plate. They then ran forward at a self-selected speed, planted one foot in the force plate, and then backpedaled back to the original starting position. For the 45° run cut task, the subject began approximately 12 feet away from the force plate. They then ran forward at a self-selected speed, planted one foot in the force plate, and pushed off the planted foot at a 45° angle to finish running for 3–4 more strides. More detailed descriptions and lab setup for each task are defined in [31]. Participants completed multiple repetitions of each task until two successful trials were completed on each leg to the satisfaction of the research staff. Criteria for success included landing without loss of balance (single-leg hop) and planting a single foot fully inside the force plates while changing directions (deceleration and run cut) [31].

### 2.4. Data Processing

Motion capture data were processed in Vicon Nexus 2.11 software. Marker trajectories were filtered using a Woltring filter with a predicted mean square error of 10 mm^2^ [32], and force plate data were filtered using a fourth-order lowpass Butterworth filter with a cutoff frequency of 16 Hz [33]. Segment orientation relative to a global coordinate system as well as joint angles and moments were computed using a custom 6-degree-of-freedom model in MATLAB 2022a (MathWorks, Natick, MA, USA). Internal lower extremity joint kinetics were computed using inverse dynamics and normalized to body mass (in kilograms). For the immediate analysis, peak KAM was collected by extracting the coronal plane knee moment within the task phase of interest.

Task events were identified using a custom MATLAB code based on predefined criteria. Each task had a specific phase of interest for this study: landing phase for the single-leg hop (initial force plate contact to lowest point of the sacrum), and plant phase for the deceleration and run cut tasks (initial force plate contact to lift-off from force plate). KAM of the plant leg as well as tri-axial acceleration of the thigh, shank, pelvis, and trunk were extracted from the phase of interest from each trial. Specifically, maximum KAM and maximum segment acceleration in each plane were extracted, along with the timing of each peak. The timing of each peak acceleration and peak KAM were normalized by conversion to a percentage of the task phase.

### 2.5. Data Analysis

For each task, one representative trial of the two repetitions completed was selected based on best performance (longest hop distance or fastest entry velocity). Correlations between peak KAM and maximum segment accelerations were analyzed across all participants using the representative trial selected for each task. Specifically, Shapiro–Wilk tests of normality were performed. Given non-significant results, Pearson correlations were performed. Correlation coefficients less than or equal to 0.35 were considered weak, between 0.36 and 0.67 were considered moderate, and between 0.68 and 1.0 were considered strong [34]. The mean difference between the timings of peak KAM and peak segment acceleration were analyzed using paired t-tests, with a statistically significant mean difference indicating that peak KAM and peak segment acceleration did not occur at the same time. A positive time difference indicated that peak segment acceleration occurred before peak KAM, while a negative time difference indicated that peak KAM occurred prior to peak segment acceleration. Significance level (α) for all statistical tests was set at 0.05. Statistical analyses were performed using SPSS (IBM Corp., Armonk, NY, USA).

## 3. Results

Although all participants completed all three tasks, a few participants were removed from analysis, as force plate data were not obtained during testing: six from single-leg hop, one from deceleration, and four from run cut. Mean values for peak KAM and segment accelerations for all three tasks are provided in Table A1 of Appendix A. Pearson correlation coefficients between peak KAM and peak segment accelerations for all three tasks are presented in Table 2. One significant moderate correlation was identified for the single-leg hop task between peak KAM and peak posterior shank acceleration (r = 0.547, *p* = 0.043). Additionally, the average time of peak KAM occurrence and time of peak posterior shank acceleration occurrence were not significantly different (*p* = 0.604), indicating these peaks occurred at approximately the same timepoint during the hop task. No other peak accelerations for any segment in any direction were significantly associated with peak KAM during the single-leg hop task.

For the deceleration task, moderate positive correlations between peak inferior acceleration of the thigh and shank with peak KAM were observed (thigh: r = 0.459, *p* = 0.027; shank: r = 0.443, *p* = 0.034), while a moderate inverse correlation between peak inferior acceleration of the trunk and peak KAM was observed (r = −0.455, *p* = 0.029). Additionally, peak KAM was moderately correlated with peak anterior acceleration of the thigh (r = 0.604, *p* = 0.002) and moderately correlated with peak anterior acceleration of the shank (r = 0.424, *p* = 0.044), but these peak accelerations did not occur on average at the same time as peak KAM (thigh: *p* = 0.026, shank: *p* = 0.006).

For the run cut task, a moderate inverse correlation between peak KAM and peak anterior (r = −0.513, *p* = 0.035) and inferior (r = −0.501, *p* = 0.040) trunk acceleration was observed. These peak accelerations occurred on average at the same time as peak KAM (anterior: *p* = 0.057, inferior: *p* = 0.123). Additionally, a moderate positive correlation was measured between peak KAM and peak anterior pelvic acceleration (r = 0.486, *p* = 0.048). The only coronal plane peak acceleration significantly associated with peak KAM across all tasks was lateral shank acceleration during the run cut task, which exhibited a moderate positive correlation (r = 0.573, *p* = 0.016).

## 4. Discussion

The purpose of this study was to investigate potential relationships between peak segment accelerations of the trunk and lower extremities and KAM during sport-specific movements. Overall, significant associations were found across the single-leg hop, deceleration, and run cut tasks. Peak KAM was found to be associated with posterior shank acceleration during the single-leg hop task. Additionally, significant associations with peak KAM were found with trunk, thigh, and shank accelerations during the deceleration task and with trunk, pelvis, and shank accelerations during the run cut task.

During the single-leg hop task, higher peak KAM was associated with higher posterior shank acceleration. During hop task landings, smaller knee flexion angles and increased tibial anterior shear forces (i.e., stiffer landings) have been associated with higher ACL loading [35]. Furthermore, Li et al. reported that participants who were instructed to perform a single-leg drop landing with a ‘soft landing’ demonstrated reduced peak vertical ground reaction forces compared to participants landing with their natural landing strategy [36]. The correlation between peak KAM and peak posterior shank acceleration found in this current study is thought to be a result of a stiffer landing strategy, resulting in a higher ground reaction force and causing the shank to accelerate posteriorly. This is supported by the nonsignificant difference in the timing of peak KAM and peak posterior shank acceleration, which indicates that the two events occurred at approximately the same time during the landing phase.

For the deceleration task, higher peak inferior acceleration of the thigh and shank were associated with higher peak KAM. It is suggested that an effective breaking strategy involves not only the penultimate foot contact but also the preceding foot contacts to help distribute the breaking forces over multiple gait cycles. If the athlete is unable to dampen the breaking force, then the plant foot may be subject to higher knee joint loads, thereby resulting in a rapid inferior acceleration of the thigh into the shank and the shank into the ground [37,38,39]. Conversely, a higher peak inferior acceleration of the trunk was found to correlate with lower peak KAM during the deceleration task. This inverse correlation may be a result of the athlete’s ability to quickly drop their center of mass to efficiently decelerate. Sigward et al. cited positions of a lower vertical and more posterior center of mass relative to the lead leg breaking foot as important components to improve stability during deceleration [13]. Lastly, higher peak anterior acceleration of the thigh and shank were associated with higher peak KAM. Given that this correlation did not occur at the same time point as peak KAM, this may be a result of increased running speed. This is consistent with the literature, which reports increased knee joint loading with faster running velocities during change-of-direction maneuvers [11,40]. Specifically, Vanrenterghem et al. reported increased knee valgus loading during 45° sidestep cuts with faster, compared to slower, running velocity [40].

During the run cut task, higher KAM was associated with lower peak anterior and inferior trunk acceleration. Similar to the deceleration task, this may relate to the athlete’s breaking strategy and their ability to quickly and efficiently lower their center of mass as they decelerate to change directions. This theory is further supported by the association found between higher peak KAM and higher peak anterior pelvic acceleration, which may again be a result of an inefficient braking strategy and a failure to drop the hips down and back [41]. Jones et al. highlighted similar strategies and reported a significant correlation between average horizontal ground reaction force during penultimate foot contact and KAM during a 90° cutting task (r = −0.569, *p* = 0.006) [42]. Additionally, in this current study, higher lateral shank acceleration was associated with higher KAM during the run cut task. Planting with a wide cut width during a change-of-direction maneuver has been associated with higher knee joint loads [11,37]. Jones et al. also reported a significant correlation between lateral leg plant distance and peak KAM during a 90° cutting maneuver in elite female soccer players [37]. Similarly, Kristianslund et al. identified increased cut width as one of the strongest predictors of KAM in female handball players [11]. As the athlete rapidly plants the foot further away from the body (i.e., a more lateral position), a higher lateral shank acceleration would be expected. It is important to note that each peak segment acceleration found to be significantly correlated with peak KAM during the run cut task was found to occur at approximately the same time as peak KAM during the plant phase.

This study has several limitations that should be noted. First, as this was a pilot study, the sample size was small and limited to young adult participants. Thus, findings cannot be generalized to youth athletes, and additional data must be collected to further investigate the findings of this current study. Future studies should aim to determine the association of acceleration data with KAM in youth athletes, given their higher risk of sport-related knee injury, and this study should be repeated with a larger sample size. Next, a planned cutting task was used in this study. It should be noted that unplanned cutting tasks have been shown to result in higher knee joint loads [43]. Additionally, the thigh and shank IMUs were placed on the rectus femoris and tibialis anterior muscle bellies, respectively, in order to collect electromyography data for a separate study. More optimal sensor placement may exist for risk assessment in a clinic setting. Next, no warm-up protocol was used prior to testing procedures for the subjects in this study. Although no injuries occurred during testing, a standardized warm-up protocol further reduces the injury risk for study participants in similar studies. Finally, given that the primary aim of this study was to evaluate the association between peak KAM and segment accelerations, future analyses should investigate the relationship between segment accelerations and additional biomechanical variables to better understand the utility of wireless accelerometers in identifying a variety of movement strategies and potential deficiencies.

## 5. Conclusions

This study demonstrated moderate correlations between peak KAM and trunk, pelvis, thigh, and shank segment acceleration, derived from wireless accelerometers during common athletic tasks. These findings provide preliminary support for the use of wearable IMUs as a surrogate to identify peak KAM. Further work is needed to validate the ability of accelerometers to identify high KAM in a larger sample of youth athletes and to determine their ability to predict ACL injury.

## Figures and Tables

**Table 1:** Functional task verbal instructions.

Functional Task	Verbal Instruction
Single-Leg Hop	Start by standing on one leg at the starting line. Then, hop forward as far as you can, landing on the same leg. You must stick the landing for at least 3 s.
Deceleration	Run forward and plant your foot in the square on the floor, and then backpedal back to the starting line. Run and backpedal as fast as you feel comfortable.
Run Cut	Run as fast as you feel comfortable toward the cone. Land with your foot inside the square on the floor before cutting at a 45° angle and continue running for 3–4 strides before stopping.

**Table 2:** Correlation results of peak segment acceleration vs. peak KAM with timing differences.

	Single-Leg Hop		Deceleration		Run Cut	
	r (*p*-Value)	Time Diff. (SD)	r (*p*-Value)	Time Diff. (SD)	r (*p*-Value)	Time Diff. (SD)
**Trunk**						
Lateral	−0.303 (0.292)	**−18.8** (**30.1**)	0.037 (0.866)	−19.0 (54.5)	0.168 (0.519)	−13.4 (55.2)
Medial	0.042 (0.888)	5.6 (35.8)	−0.024 (0.912)	−9.8 (33.1)	−0.024 (0.928)	−6.5 (34.2)
Anterior	0.137 (0.640)	10.4 (37.8)	−0.081 (0.714)	**−23.8** (**54.9**)	**−0.513** (**0.035**)	−16.2 (32.7)
Posterior	0.159 (0.587)	−3 (34.4)	0.282 (0.193)	−14.1 (47.1)	0.433 (0.082)	**23.9** (**44.6**)
Superior	−0.155 (0.596)	**−17.6** (**28.4**)	−0.218 (0.317)	4.2 (36.1)	0.168 (0.519)	6.1 (35.8)
Inferior	0.011 (0.969)	**36.3** (**27**)	**−0.455** (**0.029**)	11.0 (46.1)	**−0.501** (**0.040**)	21.1 (53.3)
**Pelvis**						
Lateral	−0.516 (0.059)	**−25.3** (**23.8**)	0.177 (0.419)	13.9 (34.4)	0.199 (0.443)	**31.9** (**35.0**)
Medial	−0.276 (0.340)	5.9 (25.9)	0.075 (0.733)	14.2 (37.4)	0.036 (0.890)	11.7 (50.5)
Anterior	−0.340 (0.234)	**20.4** (**38.9**)	−0.054 (0.808)	3.4 (57.6)	**0.486** (**0.048**)	−12.5 (59.3)
Posterior	−0.205 (0.481)	11.6 (26.5)	−0.104 (0.638)	−4.2 (48.6)	−0.063 (0.809)	**28.8** (**37.9**)
Superior	−0.139 (0.636)	1.2 (26.9)	0.039 (0.859)	11.7 (35.0)	0.129 (0.621)	12.0 (42.5)
Inferior	−0.444 (0.112)	**35.3** (**27.5**)	−0.002 (0.992)	**26.3** (**33.6**)	−0.115 (0.661)	**42.5** (**36.1**)
**Thigh**						
Lateral	0.258 (0.394)	−5.1 (28.2)	0.188 (0.391)	**17.2** (**30.3**)	−0.195 (0.454)	**26.5** (**38.6**)
Medial	−0.185 (0.544)	**−17.5** (**26.5**)	0.029 (0.894)	7.9 (31.9)	0.345 (0.176)	4.9 (47.5)
Anterior	0.145 (0.637)	4.5 (25.4)	**0.604** (**0.002**)	**20** (**40.2**)	0.315 (0.218)	19.5 (47.2)
Posterior	0.200 (0.512)	−3.1 (26.6)	−0.218 (0.318)	−18 (45.9)	−0.195 (0.453)	**29.9** (**32.3**)
Superior	−0.129 (0.674)	8.1 (23.8)	0.196 (0.369)	**20.8** (**30.7**)	0.122 (0.641)	23.2 (47.8)
Inferior	0.147 (0.632)	−7.1 (28.5)	**0.459** (**0.027**)	12.8 (38.5)	0.250 (0.332)	**29.6** (**39.5**)
**Shank**						
Lateral	−0.304 (0.290)	−4.3 (26.9)	0.040 (0.857)	5.3 (39.9)	**0.573** (**0.016**)	17.8 (42.4)
Medial	0.349 (0.221)	**18.3** (**27.9**)	−0.291 (0.178)	11.1 (43.8)	0.299 (0.244)	−8.6 (61.8)
Anterior	0.160 (0.585)	−5.2 (43.7)	**0.424** (**0.044**)	**21.5** (**33.9**)	0.081 (0.756)	**−25.4** (**37.3**)
Posterior	**0.547** (**0.043**)	−3.4 (27.5)	−0.187 (0.392)	13.6 (31.6)	−0.015 (0.954)	14.9 (39.8)
Superior	0.036 (0.903)	8.2 (30.2)	−0.189 (0.388)	**21.8** (**31.3**)	0.070 (0.789)	3.2 (52.1)
Inferior	−0.085 (0.772)	−4.9 (23.4)	**0.443** (**0.034**)	16.7 (40)	0.300 (0.242)	**29.4** (**35.4**)

Note: r represents the Pearson correlation coefficient for the association between the peak acceleration measure and peak KAM. Time Diff. represents the mean difference between the timepoints at which the peak acceleration measure and peak KAM occurred, with a positive difference indicating that peak acceleration occurred before peak KAM. Time Diff. is reported as a percentage of the task phase, and standard deviation is provided for each mean difference in parentheses. Lastly, significant correlations are indicated with bolding of the r, while significant differences in timing are noted with bolding of the Time Diff.

## Data Availability

The data presented in this study are available on request from the corresponding author.

## Appendix A

**Table A1:** Peak KAM and Peak Segment Acceleration.

	Single-Leg Hop		Deceleration		Run Cut	
	**Peak KAM (NM/kg)**	**SD**	**Peak KAM (NM/kg)**	**SD**	**Peak KAM (NM/kg)**	**SD**
	0.66	0.29	0.39	0.24	0.82	0.45
	**Peak Acceleration (m/s^2^)**	**SD**	**Peak Acceleration (m/s^2^)**	**SD**	**Peak Acceleration (m/s^2^)**	**SD**
**Trunk**						
Lateral	2.07	3.24	1.71	1.41	−0.18	2.06
Medial	5.05	3.21	4.02	1.67	12.35	4.37
Anterior	−0.57	3.92	1.39	3.13	2.31	2.64
Posterior	8.31	4.23	6.85	4.40	6.72	4.52
Superior	24.49	4.16	20.57	3.85	24.48	4.05
Inferior	−0.85	2.88	0.06	2.13	1.28	3.59
**Pelvis**						
Lateral	13.20	7.14	5.19	3.07	7.88	3.14
Medial	22.06	5.36	10.39	4.52	11.46	5.33
Anterior	−2.40	3.95	−0.46	1.47	1.57	2.36
Posterior	22.65	3.13	12.01	2.60	12.86	4.14
Superior	25.39	4.76	18.85	3.70	23.47	3.69
Inferior	1.52	2.40	0.95	3.32	3.31	3.37
**Thigh**						
Lateral	12.80	7.40	8.14	3.11	13.50	6.24
Medial	11.36	5.26	8.67	2.94	11.66	4.41
Anterior	9.64	5.01	15.88	5.86	18.57	7.85
Posterior	14.75	7.01	8.34	4.17	13.94	4.63
Superior	43.36	15.58	31.6	7.10	34.67	9.27
Inferior	6.32	7.25	0.35	4.91	7.43	7.47
**Shank**						
Lateral	21.51	6.61	14.28	4.34	19.84	4.89
Medial	9.00	5.80	9.42	5.95	10.09	8.95
Anterior	−1.63	6.41	12.87	6.22	11.77	10.94
Posterior	24.78	8.00	15.32	4.18	20.71	4.85
Superior	37.21	9.94	30.47	5.10	34.91	7.81
Inferior	6.18	7.11	1.55	6.03	10.14	6.37

Note: Peak KAM and peak segment acceleration values are reported as the mean ± SD across all participants. Negative values for peak segment acceleration indicate that no acceleration occurred in that direction during the task phase of interest, and therefore the magnitude of the reported peak acceleration represents the minimum acceleration in the opposing direction.
